# A clinical image: posterior meningocele

**DOI:** 10.11604/pamj.2022.42.288.36209

**Published:** 2022-08-17

**Authors:** Anushri Kale, Bibin Kurian

**Affiliations:** 1 Department of Child Health Nursing, Smt. Radhikabai Meghe Memorial College of Nursing, Datta Meghe Institute of Medical Sciences, Sawangi (Meghe), Wardha, Maharashtra, India

**Keywords:** Posterior meningocele, neural tube, archachnoid mater, spina bifida

## Image in medicine

Posterior meningocele is a rare neural tube closure defect characterized by the herniation of a cerebrospinal fluid-filled sac, that is lined by dura and arachnoid mater, through a posterior spina bifida and covered by a layer of skin of variable thickness, which may be dysplastic or ulcerated. They are most commonly located in the lumbar or sacral region. A 1-year-old male child was brought to the outpatient department with the complaint of swelling in the back, swelling increases gradually and to the present size. After detailed history collection and physical examination, it reveals that he is having swelling in the sacral region since birth. The physician diagnosed him with posterior meningocele and hence referred them to the inpatient department for further surgical management.

**Figure 1:**
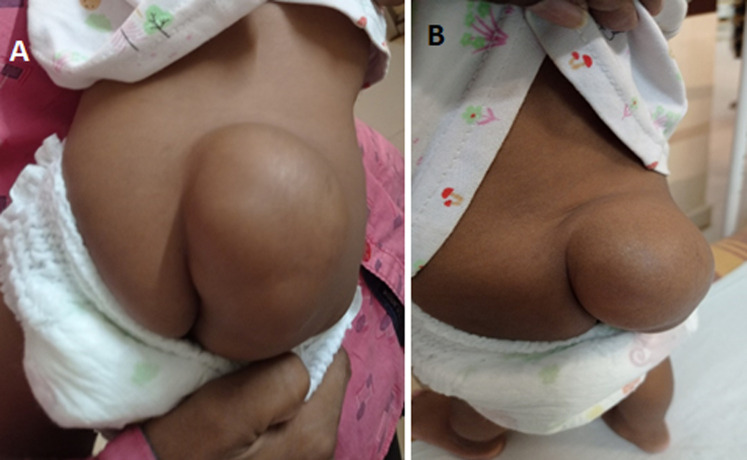
(A,B) posterior meningocele

